# MHealth and perceived quality of care delivery: a conceptual model and validation

**DOI:** 10.1186/s12911-020-1049-8

**Published:** 2020-02-27

**Authors:** Yvonne O’Connor, Pavel Andreev, Philip O’Reilly

**Affiliations:** 1 Department of Business Information Systems, University College Cork, O’ Rahilly Building, Cork, Ireland; 2 Telfer School of Management, University of Ottawa, Ottawa, Canada

**Keywords:** Mobile systems, Post-implementation, Information technology utilization, Quality of care

## Abstract

**Background:**

The objective of this research is to examine, conceptualize, and empirically validate a model of mobile health (mHealth) impacts on physicians’ perceived quality of care delivery (PQoC).

**Methods:**

Observational quasi-experimental one group posttest-only design was implemented through the empirical testing of the conceptual model with nine hypotheses related to the association of task and technology characteristics, self-efficacy, m-health utilization, task-technology fit (TTF), and their relationships with PQoC. Primary data was collected over a four-month period from acute care physicians in The Ottawa Hospital, Ontario, Canada. The self-reported data was collected by employing a survey and distributed through the internal hospital channels to physicians who adopted iPads for their daily activities.

**Results:**

Physicians’ PQoC was found to be positively affected by the level of mHealth utilization and TTF, while the magnitude of the TTF direct effect was two times stronger than utilization. Additionally, self-efficacy has the highest direct and total effect on mHealth utilization; in the formation of TTF, technological characteristics dominate followed by task characteristics.

**Conclusion:**

To date, the impact of utilized mHealth on PQoC has neither been richly theorized nor explored in depth. We address this gap in existing literature. Realizing how an organization can improve TTF will lead to better PQoC.

## Contributions to the literature

- The use of mHealth and physicians’ perceptions regarding their satisfaction and performance as it impacts upon quality of patient care is under investigated.
- A conceptual model is developed and empirically examined to understand the factors which impact physicians’ perceived quality of care (PQoC) when employing mHealth artefacts when delivering healthcare services in a hospital setting.
- Our findings demonstrate that physicians using mHealth at the point-of-care enhances their PQoC a patient receives. These findings can be used to inform implementation strategies to increase the use of mHealth in hospitals.

## Background

The mobile health (mHealth) market has experienced a significant growth since 2011, with the global mHealth market estimated to grow to about $60 billion by 2020. Lee [1] argued that that many hospitals are spending vast amount of money for implementing mHealth solutions and expecting that users (i.e. physicians) will employ the technology to improve the quality of care patients receive at the point-of-care. Concurrently, hospitals face increasing demands to participate in a wide range of quality improvement activities [2, 3] by streamlining their processes in order to deliver high quality and safe care [4, 5]. Instant real-time access to data at the point-of-care is causing a paradigm shift in how physicians deliver healthcare, making services more streamlined and cost effective [6].

Despite the wide endorsement and support for the implementation of mHealth, Rahurkar [7] argue that empirical evidence surrounding the benefits of Information Technology (IT) in health care remains to be firmly established. The limited evidence for the performance of mHealth technologies may be attributable to a lack of appropriate evaluation frameworks [8–10], with Boudreaux et al., [11] arguing that there exists no evalutation method which is mHealth specific. While some attempts have been made by various researchers [12–16] to evaluate mHealth, this work has predominantly focused on reviewing and reporting the adoption of mHealth technologies at early phases of implementation. The lack of evaluation across the mHealth field, primarily in post-adoption stages of implementation, may be perceived as a major weakness of the mHealth domain [9, 10]. Therefore, for mHealth to be truly valuable and have a positive impact on quality of healthcare delivery, the IT artefact must be utilized post-adoption [17, 18]. Furthermore, Goodhue and Thompson [19] argue that the capabilities of the IT must match the tasks that the user must perform. Serrano and Karahanna [20] argue that the role of the user in influencing successful task performance has not been explored in-depth within a healthcare setting. Indeed, there has been a dearth of research focused on exploring an mHealth specific evaluation framework [11, 18] in a post-adoptive scenario, specifically in a hospital setting [21].

### MHealth and quality of care delivery

MHealth, for the purposes of this study, refers to the application of mobile device/s and medical/clinical application(s) run on the device by physicians in a hospital domain, for communication, collaboration, and coordination of the physician’s healthcare delivery daily activities in hospital premises including diagnosis, treatment, and disease management (adapted from [22]).

It is purported [23] that based upon ones experiences with using a new technology and having new information to hand, an individual forms a post-adoptive perception, which may potentially deviate from prior beliefs. Indeed, they note that such deviation will both impact user’s ongoing perceived usefulness of the technology and influence user satisfaction, which will influence one’s intention to continue to utilize the technology. While mHealth has promised major benefits at the national, regional, community, and individual levels, insufficient attention has been paid to the perceived quality of care delivery (PQoC) which can be obtained from using technological artefacts [7, 24, 25]. Research has established that negative outcomes from IT utilization are possible [24, 26]. Therefore, if the utilization of mHealth is perceived to potentially harm the quality of care a patient receives, then it is unsafe for delivering healthcare services [27].

Various indicators for quality of care exist; namely, structure, process, and outcome of care delivery [28]. Quality of care is often measured from two perspectives; perceived and actual [24, 29], across several potential stakeholders (e.g. patients, physicians, administrative staff). Actual quality of care “relates to the interaction between health-care providers and patients and the ways in which inputs from the health system are transformed into health outcomes” [30]. Conversely, PQoC, in this study, is the physicians’ perceptions regarding their satisfaction and performance as it impacts upon quality of patient care. Examining actual quality of care is extremely difficult to attribute wholly to the IT artefact in a complex multifaceted environment and requires an unambiguous evidence base [30]. Furthermore, Serrano and Karahanna ( [20] p.616) purport that the impact of mHealth utilized by physicians on PQoC (consultation delivery, in the context of Serrano and Karahanna’s paper), has not been explored in depth. Therefore, the research addresses this call for research and puts forward the research question of *what are the impacts mHealth artefacts have on physicians’ perceived quality of care delivery in a hospital setting*. To answer this question, we defined two main research objectives: 1) to examine and conceptualize a model of mHealth impacts on PQoC and 2) empirically validate this model.

## A perceived quality of care delivery model: theoretical underpinnings and model development

Task-Technology Fit (TTF) reveals the association between IT and individual performance and is based on the premise of ‘fit’. ‘Fit’ has been widely utilized and is associated with performance. For example, MIT’s 90’s model is underpinned by the theory of fit [31, 32] and argues that fit contributes to high performance [31–33]. Optimal performance occurs only if there is a tight fit (alignment) among the domains of strategy, structure, management processes, individual roles and skills and technology [31].

TTF operates at an individual level of analysis with the position that IT is more likely to have a positive impact on individual performance and be utilized if the capabilities of the IT match the tasks that the user must perform [19, 34]. It consists of five constructs namely, task characteristics, technology characteristics, task-technology fit, performance impacts and utilization. TTF theory as a theoretical lens for understanding the performance of electronic health (eHealth) technologies has previously been explored [35]. Specifically, Chiasson et al. [35] answers the call for research by Furneauz [36] to understand the effect of user performance on utilization and the association between the use of effective technology and user performance. They found TTF to be a useful theory for exploring IT in healthcare and illustrated the positive association between use and performance.

However, in the context of performance, the association between the utilization of a technology and the PQoC (as a performance outcome) has not been investigated. It is noted [4, 37] that there is scant empirical evidence of the impact which healthcare information systems have on the quality of care. Towards deriving an understanding of same, a conceptual model is developed to explore physicians’ view which mHealth has on PQoC. Specifically, a conceptual model is developed (based on the technology to Performance chain model) to focus on PQoC as the dependent variable.

### Hypotheses development

Researchers have found that mobile technologies impact performance of mobile workers and promote efficiency [38–41]. When IT artefacts are embedded within an individual’s work practice, then they must facilitate the accomplishment of their work [42, 43]. System usage for this study is defined as the degree to which mHealth is incorporated into the users’ (i.e. physicians) work processes or tasks. The concept of usage focuses on *incorporation* and comprises routine, feature and value-adding use [44]. Feature use refers to the extent to which physicians use the mHealth features/functionality to complete any given task (adapted from [45]). Routine use is important in this study as this research is examined in a post-adoptive scenario. Therefore, routine use refers to the extent to which a healthcare physician tends to use mHealth automatically (adapted from [46]). Finally, value-adding use is the extent by which physicians capture “the additional (none-core, non-automated and/or non-compulsory) use by the user conducted to enhance the output or impact” ( [44] p.6).

Studies show that mHealth usage by physicians assists with facilitating decision support and medication safety in terms of prescribing and dispensing at the point-of-care [47, 48], thereby increasing diagnoses while decreasing missed diagnoses. Similarly, it was found [25] that hospital implementation of Health IT was positively associated with activities intended to improve patient care quality. Therefore, it is hypothesized that:

#### Hypothesis 1

Physician’s perceive that the Quality of Care delivered to their patients is positively impacted by the mHealth’s alignment with the task at hand (TTF).

#### Hypothesis 2

Physician’s perceive that the Quality of Care delivered to patients is positively impacted by physician’s utilization of mHealth.

#### Hypothesis 3

Physician’s utilization of mHealth is positively impacted by its alignment to the task at hand.

While technology is typically aligned with organizational structures, it is not aligned with care coordination i.e. healthcare physician tasks [2]. Task characteristics, in this study, are defined broadly as the actions taken by physicians and include flexibility, protocol adherence, and time criticality [2, 18]. Due to the complex nature associated with the delivery of healthcare services, flexibility in workflows is clinically pertinent. The flexibility of a process is its ability to deal with both foreseen and unforeseen change [49]. Patient care in most environments is by its very nature a mobile experience [50]. Common problems which arise in healthcare settings include unavailable medical information at the time of treatment, replication of test results, protocols not being followed and prescription of incorrect medications [2]. Therefore, we propose the following hypothesis:

#### Hypothesis 4–1

Physicians perceptions of Task Technology alignment will be positively impacted by Healthcare task characteristics.

#### Hypothesis 4–2

Healthcare task characteristics impacts mHealth utilization by physicians in a healthcare setting.

Research argues that technological resources are required for system usage [51]. Technology (i.e. mobile) characteristics refer to specific features, functionality, or usability of a technology that can affect its usage by target users [52]. It is argued that the implementation of any eHealth technology must live up to its fullest potential in real-world conditions and circumstance [18, 53], therefore having real world value. Existing research argues that physicians may be reluctant to utilize some IT technologies because they may fear it will not perform reliably or possess insufficient functionality for users to perform tasks. Therefore, we hypothesize:

#### Hypothesis 5–1

Physician perceptions of Task Technology alignment will be positively impacted by mHealth characteristics.

#### Hypothesis 5–2

The mHealth characteristics impact upon its use by physicians in a healthcare setting.

Research [54, 55] argues that self-efficacy tailored to an IT artefact is an important determinant of a variety of user perceptions of technology. As a result, self-efficacy has received considerable empirical support in a vast array of papers spanning both pre-and post-adoption research studies. Self-efficacy is defined as the degree to which an individual’s perceives their ability to utilize mHealth in the accomplishment of a task (adapted from [56]). Shaw and Manwani [57] found that physicians with high self-efficacy had greater potential to extensively use the vast array of features offered by a technology. Moreover, it is argued [58–61] that individuals with high self-efficacy tend to perform well when conducting a variety of tasks using IT. Pierce et al. [62] found that feelings of self-efficacy encourage individuals to explore and manipulate the environment within which they work and to feel a sense of empowerment. Therefore, it is hypothesized that:

#### Hypothesis 6–1

Physician perceptions of Task Technology alignment will be positively impacted by their perceived ability to utilize mHealth.

#### Hypothesis 6–2

Physicians perception of their ability to employ mHealth positively impacts utilize mHealth.

**Figure 1:**
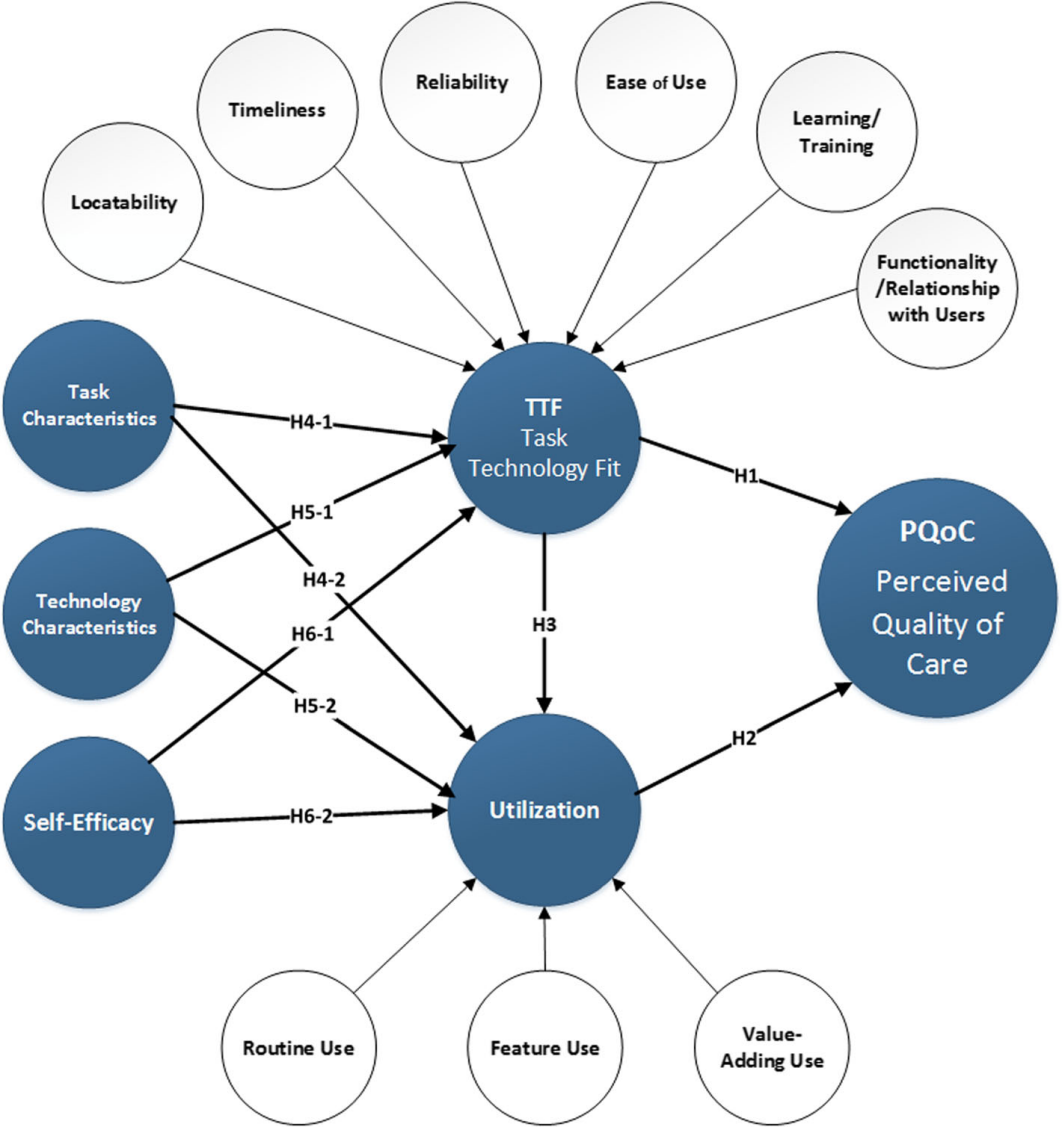
**Fig. 1** Conceptual Model

Figure 1 presents the conceptual model employed in this study. The next section will discuss how we operationalized this model (Fig. 1).

## Methods

To test the conceptual model (Fig. 1) an observational quasi-experimental one group posttest-only design was employed. The self-reported data was collected by employing a survey and distributed through the internal hospital channels to physicians who adopted iPads for their daily activities. The online survey questionnaire was constructed using indicators already validated in existing research (see Additional file 1). The survey instrument was piloted to ensure content and construct validity. Over 20 medical experts (professionals who work in healthcare delivery and actively utilize mHealth as a part of their daily work related activities) in the US and Ireland participated in the pilot study. Following this, the questionnaire was refined before launching the survey.

Data was collected over a four-month period, in 2012, from physicians in an acute care context within The Ottawa Hospital, Ontario, Canada. The Ottawa Hospital (TOH) made over 3000 iPads, incorporating a mobile Electronic Medical Record application, available for use by physicians. TOH is one hospital spanning three sites (Civic, Riverside, and General Campuses) and has been using mHealth at the point-of-care for numerous years. The survey was distributed via email (an URL link) to physicians in TOH, Canada. No hard copies were distributed to the potential participants since all members of the TOH staff had Internet access.

One major types of bias that is often associated with web surveys is common method variance bias [63]. To overcome the former, several reversed-scored items were used to reduce sign rating problems associated with Likert scales [63]. See Additional file 1 for a description of the items used as part of the questionnaire.

Participants using mHealth within their daily practices for 6 months or more was eligible for the study. A total of 157 responses were obtained from various physicians via the administration of an online survey (871 physicians in total at the time of data collection), yielding a response rate of 18%. Not all of the 871 physicians were available/active during the data collection period of time, which may have impacted the response rate. Noteworthy, it is argued that physicians are often a group with low survey response rates [64]. Nevertheless, 157 responses were cleaned for missing values and 102 complete responses were used for data analysis. While research [65] argues that missing values affects directory of taking the decision, G*Power (version 3.1.2) was used to conduct power analysis and to establish whether the sample size is sufficient. The analysis revealed a power value close to one indicating that the sample size of 102 is sufficient as per Cohen [66]. A key benefit of using Partial Least Square (PLS)- Structural Equation Modeling (SEM) is that it accommodates the use of small sample sizes giving that the ten [10] times rules is met. The 10 times rule depicts that a sample size should be equal to 10 times the largest number of formative indicators used to measure a particular construct, or 10 times the largest number of paths directed at a construct in the model [66]. Our sample size satisfies both requirements.

Structural Equation Modeling (SEM) was used for the hypotheses testing and data analysis. The PLS (SEM) approach, which uses component-based estimation was chosen since it allows simultaneous examination of both the measurement and the structural models. The measurement (outer) model portrays the relationships between a construct and its associated measurement items whereas the structural (inner) model represents direct and indirect unobservable relationships among constructs [67, 68]. In addition, the PLS approach, in contrast to covariance-based SEM, allows testing of the relationships in the model with less restrictive requirements and relatively small sample sizes. PLS is also considered very appropriate for exploratory studies and for testing theories at earlier stages of development [69], and it is highly suitable for prediction-oriented research [70].

**Figure 2:**
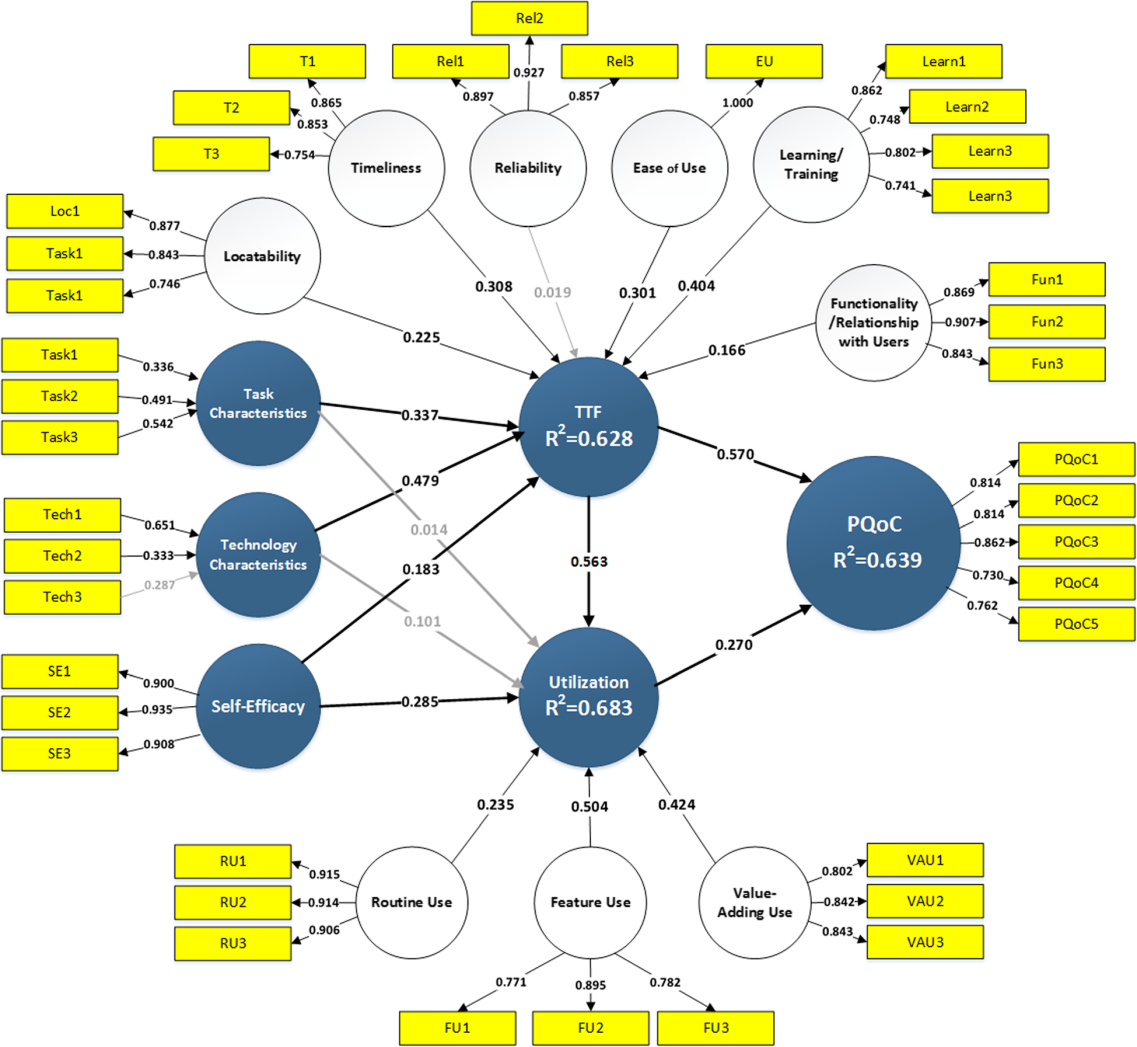
**Fig. 2** Evaluation of measurement and structural models

The evaluation process of the PLS path model with mixed formative and reflective constructs involves two steps [70–75]. Step 1 involves the testing of the quality of the measurement (outer) models of first-order constructs used in the first stage as well as other endogenous and exogenous constructs. After this we assess the appropriateness of the high order constructs. The research model includes two second-order constructs TTF - task technology fit and Utilization (Fig. 2). We followed the recommendations provided by Becker et al. [76] for repeated indicators, two-stages, and hybrid approaches for estimation hierarchical second-order constructs. As Step 1 was successful and latent constructs were found to be reliable and valid, Step 2, which necessitates the assessment of the structural (inner) model, was conducted [70, 75]. SmartPLS 3.2.6 was employed for the PLS model assessment.

## Results

After excluding 55 responses from the 157 received, 102 surveys were usable for data analysis. This cohort represented 59 males and 43 females, with ages ranging from 18 to 25 years (*n* = 2); 26–40 years (*n* = 58); 41–55 years (*n* = 25); 56–65 years (*n* = 14) and > 65 years (*n* = 3). Attending Physicians accounted for 53% of the responses (*n* = 54), with Residents/Fellows completing the remainder of 47%.

The reflective measurement constructs of the research model were assessed according to the established criteria to assess PLS models with reflective constructs (e.g., [67, 70, 73]). The research model includes six first-order reflective constructs of higher-order construct Task-Technology Fit, three first-order reflective constructs of a second-order construct Utilization, one exogenous reflective construct Self-Efficacy, and one endogenous PQoC (Fig. 1). Hence 10 (ten) reflective measurement models were assessed for reliability and validity. Table 1 demonstrates Individual reliability of the indicators - the magnitudes of all indicators are above this lower limit of 0.707, with the lowest value of 0.731 and majority of values above 0.8.

**Table 1:** Construct reliability and convergent validity

Constructs	Indicator	Indicator Reliability/Convergent Validity				Internal Consistency Reliability	
		Loading	T-Stat	*p*-value	AVE	Cronbach’s α	Composite Reliability
Performance (PQoC)	PQoC1	0.815	26.58	0.00	0.636	0.856	0.897
	PQoC2	0.814	19.16	0.00			
	PQoC3	0.861	23.83	0.00			
	PQoC4	0.731	12.11	0.00			
	PQoC5	0.761	14.38	0.00			
Self-efficacy (SE)	SE1	0.900	33.41	0.00	0.836	0.902	0.939
	SE2	0.934	34.19	0.00			
	SE3	0.908	26.53	0.00			
Learning/Training (Learn)	Learn1	0.862	33.79	0.00	0.624	0.802	0.869
	Learn2	0.748	10.41	0.00			
	Learn3	0.802	17.61	0.00			
	Learn4	0.741	11.49	0.00			
Locatability (Loc)	Loc1	0.877	25.04	0.00	0.679	0.768	0.863
	Loc2	0.843	13.90	0.00			
	Loc3	0.746	9.08	0.00			
MHealth Reliability (Rel)	Rel1	0.897	36.69	0.00	0.799	0.874	0.923
	Rel2	0.927	44.36	0.00			
	Rel3	0.857	25.90	0.00			
Relationship with Users/Functionality (Fun)	Fun1	0.869	31.02	0.00	0.763	0.844	0.906
	Fun2	0.907	40.68	0.00			
	Fun3	0.843	20.86	0.00			
Timeliness (T)	T1	0.865	20.61	0.00	0.681	0.764	0.865
	T2	0.853	22.01	0.00			
	T3	0.754	9.52	0.00			
Value-Adding Use (VAU)	VAU1	0.802	18.19	0.00	0.686	0.771	0.868
	VAU2	0.841	19.74	0.00			
	VAU3	0.843	21.82	0.00			
Feature Use (FU)	FU1	0.771	12.57	0.00	0.669	0.752	0.858
	FU2	0.895	45.54	0.00			
	FU3	0.782	10.91	0.00			
Routine use (RU)	RU1	0.915	42.60	0.00	0.832	0.899	0.937
	RU2	0.914	43.12	0.00			
	RU3	0.906	30.64	0.00			

Table 1 illustrates that all manifest items are reliable for all reflective constructs, demonstrating internal consistency reliability since both parameters (i) Cronbach’s α and parameter (ii) composite reliability have high values (all values are above 0.752), with the required value being above 0.7 [70]. The validity test of the reflective constructs examines the *convergent validity* and the *discriminant validity*. Average Variance Extracted (AVE) for all constructs is higher than 0.5, which indicates sufficient convergent validity (Table 1).

We tested discriminant validity with three approaches: Fornell-Larker criterion analysis (See Table 2), cross-loadings (see Additional file 2), and assessing the heterotrait-monotrait ratio (HTMT) of the correlations (See Table 3).

**Table 2:** Fornell-Larker criterion analysis

	FU	Learn	Loc	Rel	PQoC	Fun	RU	SE	T	VAU
FU	**0.818**									
Learn	0.357	**0.790**								
Loc	0.465	0.307	**0.824**							
Rel	0.273	0.337	0.478	**0.894**						
PQoC	0.455	0.708	0.363	0.379	**0.798**					
Fun	0.376	0.366	0.507	0.644	0.507	**0.873**				
RU	0.466	0.427	0.490	0.529	0.646	0.589	**0.912**			
SE	0.475	0.295	0.536	0.496	0.369	0.440	0.535	**0.914**		
T	0.330	0.352	0.145	0.249	0.444	0.288	0.440	0.168	**0.825**	
VAU	0.464	0.632	0.409	0.276	0.613	0.359	0.571	0.485	0.456	**0.828**

**Table 3:** Heterotrait-monotrait ratio

	EU	FU	Learn	Loc	Rel	PQoC	Fun	RU	SE	T
FU	0.469									
Learn	0.490	0.424								
Loc	0.487	0.618	0.359							
Rel	0.473	0.329	0.392	0.588						
PQoC	0.667	0.554	0.830	0.420	0.433					
Fun	0.554	0.468	0.434	0.621	0.747	0.598				
RU	0.607	0.564	0.474	0.577	0.591	0.728	0.676			
SE	0.398	0.575	0.326	0.635	0.548	0.417	0.503	0.592		
T	0.320	0.425	0.428	0.188	0.299	0.545	0.361	0.532	0.204	
VAU	0.376	0.588	0.759	0.512	0.331	0.745	0.448	0.685	0.584	0.593

The results of all tests confirm that the manifest variables (indicators) presented in the research model are reliable and valid.

### Assessment of measurement models – formative constructs

The research model includes two lower order formative constructs: Task Characteristics and Technology Characteristics and two high-order formative contract: TTF and Utilization.

For assessing multicollinearity in the formative measurement model variance inflation factor (VIF) was employed. All VIF indexes were below the critical value of 5 [75] and even lower than the more strict threshold of 3.3 [68], indicating absence of multicollinearity and supporting validations of all indicators (Table 4).

**Table 4:** Multicollinearity and indicator-validity tests

Indicator - > Construct	Weight	STD	T-Stat	*P* Value	VIF
Tech1 - > Technology Characteristics	0.651	0.121	5.396	0.000	1.251
Tech2 - > Technology Characteristics	0.333	0.167	1.997	0.046	1.367
Tech3 - > Technology Characteristics	0.287	0.165	1.742	0.082	1.576
Task1 - > Task Characteristics	0.336	0.163	2.067	0.039	1.149
Task2 - > Task Characteristics	0.491	0.168	2.926	0.003	1.207
Task3 - > Task Characteristics	0.542	0.181	2.987	0.003	1.120
Ease of use - > TTF	0.301	0.075	3.991	0.000	1.604
Learning/Training - > TTF	0.404	0.080	5.027	0.000	1.387
Locatability - > TTF	0.225	0.076	2.971	0.003	1.502
MHEALTH Reliability- > TTF	0.019	0.085	0.228	0.820	1.850
Relationship with Users/Functionality- > TTF	0.166	0.083	2.001	0.045	2.050
Timeliness - > TTF	0.308	0.097	3.190	0.001	1.197
Feature Use - > Utilization	0.235	0.093	2.518	0.012	1.379
Routine use - > Utilization	0.540	0.097	5.565	0.000	1.607
Value-Adding Use - > Utilization	0.424	0.111	3.807	0.000	1.603

For testing *indicator validity*, the t-tests of indicator weight significance, accomplished with the SmartPLS program employing the bootstrap method, revealed that almost all endogenous formative latent variables met the requirements of indicator validity. One of the indicators (Tech3) of the Technology Characteristics concept was found not statistically significant with 95% confidence but significant with 90%. This indicator must be kept in the model since it represents critical dimension of the coordination role of the technology. In second-order construct of TTF the weight of the first-order construct mHealth reliability was not statistically significant. However, this construct has also been retained in the model due to theoretical consideration.

Routine Use was found as a major Utilization factor (γ = 0.540). Value-adding Use has a high impact (γ = 0.424) and is highly important and significant for utilization of mHealth. On the other hand, Feature Use was found although statically significant but the least important factor (γ = 0.235) in the Utilization construct. Learning/Training was found to be the most important component of Task Technology Fit construct (γ = 0.404). Timeliness also play a significant role in forming this construct (γ = 0.308), followed by Ease of use (γ = 0.301), Locatability (γ = 0.225), and Relationship with Users/Functionality with (γ = 0.166). Accuracy and time criticality (Task3) was the most dominating (γ = 0.542) in constructing the Task Characteristics construct. Interdependability (Task2) took the second place (γ = 0.491) and non-routines (Task1) were the weakest source (γ = 0.336) in forming this construct. The formation of Technology Characteristics construct Mobility (Tech 1) played the most critical role (γ = 0.651) followed by Personalization (Tech 2) (γ = 0.333). Coordination (Tech 3) (γ = 0.287) was not statistically significant but remained in the model.

For assessing discriminant validity, all correlations of first-order constructs were found to be higher with their second-order constructs than with any other construct in the model. Correlations of formative indicators of two formative constructs were also found higher with their own constructs than with any other construct. Through assessing content validity, construct reliability and validity of the model, it was demonstrated that the measurement models are appropriate and valid. This analysis paved the way for the evaluation of the structural mode.

### Assessment of structural model

The central criterion for evaluating the structural model is the level of explained variance of the dependent constructs. This model explains Perceived Quality of Care Delivery (PQoC), for which the R-square was 63.9%. Also, our model explains 62.8% of TTF and 68.3% of Utilization variance (Fig. 2). The variances of all three constructs were explained at the substantial or close to substantial level according to Chin’s [74] criteria. R^2^ values of 0.67, 0.33, or 0.19 for endogenous latent variables are described as substantial, moderate, or weak ( [74] p.323).

All paths coefficients except paths connecting Task and Technology Characteristics with Utilization were found to be highly statistically significant (see Table 5 and Fig. 2).

**Table 5:** Path coefficients significance test. Direct, indirect, and total effects

	Path (Direct)	T-Stat	*P* value	Indirect	Total	T-Stat	*P* Value
Self-Efficacy - > TTF (H6–1) [+]	0.183	2.261	0.024	0.000	0.183	2.261	0.024
Self-Efficacy - > Utilization (H6–2) [+]	0.285	3.631	0.000	0.103	0.388	4.692	0.000
TTF - > PQoC (H1) [+]	0.570	6.194	0.000	0.152	0.722	12.658	0.000
TTF - > Utilization (H3) [+]	0.563	5.203	0.000	0.000	0.563	5.203	0.000
Task Characteristics - > TTF (H4–1) [+]	0.337	3.718	0.000	0.000	0.337	3.718	0.000
Task Characteristics - > Utilization (H4–2) [−]	0.014	0.180	0.858	0.190	0.204	2.379	0.017
Tech Characteristics - > TTF (H5–1) [+]	0.479	5.581	0.000	0.000	0.479	5.581	0.000
Tech Characteristics - > Utilization (H5–2) [−]	0.101	1.072	0.284	0.270	0.371	4.380	0.000
Utilization - > PQoC (H2) [+]	0.270	2.790	0.005	0.000	0.270	2.790	0.005
Self-Efficacy - > PQoC_	0.000			0.209	0.209	3.440	0.001
Task Characteristics - > PQoC_	0.000			0.247	0.247	3.526	0.000
Technology Characteristics - > PQoC	0.000			0.373	0.373	5.302	0.000

PQoC was found to be positively affected by TTF (H1 supported with β = 0.570) and Utilization (H2 supported with β = 0.270). TTF affects Utilization (H3 supported with β = 0.563). TTF was found to be positively affected by Task Characteristics (H4–1 supported with β = 0.335), Technology Characteristics (H5–1 supported with β = 0.479), and Self-efficacy (H6–1 supported with β = 0.184), and Internal Knowledge (H5 supported with β = 0.379). In addition to TTF, we found that self-efficacy also affects Utilization (H6–2 supported with β = 0.295), while task and technology characteristics do not affect Utilization (H4–2 and H5–2 not supported).

In addition to path coefficients that represent direct effects we assessed indirect and total effects of the constructs. Changes in R-square were explored to investigate the substantive impact of each independent construct on the dependent construct, carrying out the *effect size* technique by re-running PLS estimations, excluding one explaining latent construct in each run. Chin [74] proposed an effect size f^2^ categorization of PLS constructs similar to Cohen’s implementation for multiple regression: small (f^2^ = 0.02), medium (f^2^ = 0.15), and large (f^2^ = 0.35). *TTF* has a large effect on both *PQoC* and *Utilization* (with f^2^ = 0.351 and f^2^ = 0.372 accordingly). While the effect of Utilization on PQoC is small (f^2^ = 0.079). There is a large effect of Technology Characteristics on TTF (f^2^ = 0.444), while effect of Task Characteristics is medium (f^2^ = 0.243) and Self-efficacy has a small effect on TTF (f^2^ = 0.072) and medium on Utilization (f^2^ = 0.192).

For the evaluation of the predictive relevance of the structural model, the Stone and Geisser test was performed using the blindfolding procedure. *Q*^2^ reflects an index of goodness of reconstruction by model and parameter estimations. A positive *Q*^2^ > 0 provides evidence that the omitted observations (from blindfolding) were well-reconstructed and that predictive relevance is achieved, while a negative *Q*^2^ reflects absence of predictive relevance. All values of *Q*^2^ were greater than zero, indicating predictive relevance for the endogenous constructs of the research model. Table 6 shows that the Q^2^ effect size for the relationships of TTF with PQoC and Utilisation can be considered as close to medium prediction relevance. Predictive relevance of Technology characteristics with respect to TTF can be considered as between small and medium while the rest of relationships have small *Q*^2^ effect size.

**Table 6:** The effect size *Q*^2^ predictive relevance test

	PQoC	TTF	Utilisation
Task	0.00	0.03	−0.01
Technology	0.01	0.10	0.00
Self-Efficacy	−0.01	0.03	0.06
Utilisation	0.01		
TTF	0.12		0.12

## Discussion

While the TTF theory has been studied in health domains [20, 77] and even variant model has been suggested such as inclusion of self-efficacy in the model [78, 79] and feed-forward chain in the TTF theory [35], a dearth of research focuses on the impact on PQoC [20]. Towards addressing this shortcoming in existing research, this study examines the impact of mHealth on the PQoC in a post-adoptive scenario. The conceptual model was developed and empirically tested (Fig. 2). The model explains 64% of the PQoC. Furthermore, it also explains 63 and 68% respectively of the endogenous constructs (TTF and utilization). TTF was found to be the dominant construct in explaining the variance of PQoC. We can infer that in a post-adoptive scenario, TTF becomes fundamental (and a very important mediator) for PQoC. Realizing how an organization can improve TTF will lead to better PQoC.

A systematic review [80] on mHealth adoption by healthcare professionals found that perceived usefulness and ease of use, design and technical concerns, cost, time, privacy and security issues, familiarity with the technology, risk-benefit assessment, and interaction with others (colleagues, patients, and management) are the main factors to providers’ adoption behavior. Our research corroborates these findings. However, towards understanding how to improve TTF, our model tested the main components of TTF and found that physicians should keep investing in learning and training, regardless of the stages of technology adoption. Learning and Training was found to be the most critical factor in the formation of TTF. Training and implementation management, as part of the Normalisation Process Theory (NPT), is argued [81] to promote the successful implementation and integration of interventions into routine work. Timeliness and Ease of Use were found to be the second and third factors in the formation of TTF. The medical domain is one which is continuously evolving, necessitating physicians to continuously learn. By utilizing mHealth to keep up to date with the latest clinical/medical protocols, physicians are continuously striving to improve quality of care.

Research confirms that self-efficacy plays an important role for IT utilization (cf. [55, 56, 82, 83]). In a post-adoptive scenario, the findings reveal that self-efficacy is critical for utilization, and this construct has the highest direct and total effect on utilization. Interpreting this finding, there is an ongoing requirement to continuously enhance an individual’s skillset for using mHealth. The findings reveal that self-efficacy has a small effect on TTF, which is unsurprising given the fact that the mHealth is already embedded in a physicians’ work practices. In the formation of TTF, the findings further reveal that technological characteristics dominate followed by task characteristics.

In the conceptual model (Fig. 2), the alignment between task and technology (TTF) has an impact upon use. Interestingly, our findings reveal that (1) the technological characteristics of mHealth and (2) healthcare physician’s work practices have no direct impact upon utilization. Towards explaining these findings, one should consider the context of the study. Data was gathered at a post-adoptive stage, meaning that mHealth had been continuously used over an extended period of time resulting in mHealth being embedded in physicians’ work practices. It was found that there is no direct impact of task characteristics and technology characteristics on mHealth utilization, although we hypothesized these impacts. However, both these constructs have significant indirect effects (TTF is a mediator) and total effects on utilization. Therefore, at the most advanced stages of utilization direct impacts are not relevant, but TTF becomes a very important mediator.

A user’s behavior can range from stagnation in utilizing IT features to total integration of the IT in his/her work domain [84–86]. Therefore, it is important that the features/functionalities of mHealth can be adapted easily to reflect the true but constant changing working nature of physicians to complete any given task within hospitals. MHealth containing electronic pharmacopoeias (i.e. drug information), medical calculations, guideline information and administrative tasks have been identified as the most useful resources by physicians, nurses and other clinical staff [87].

In the context of understanding utilization and its constituent parts, this article decomposes utilization into routine, feature, and value-adding use; the three of which have not been collectively examined in explaining utilization. By breaking utilization into these three constituent parts, we were able to identify that routine use and value-adding use are the major utilization factors for mHealth when delivering healthcare services at the point of care. At the earliest stages of adoption routine use can be very critical. However, at a post-adoption stage, routine use, while statistically significant, is the least important factor in mHealth utilization; feature use takes the leading role. An explanation for this is that as system usage becomes repetitive and habitual, routine use emerges. This confirms existing research [53]. In the context of value-adding use, physicians take advantage of the current artefact by exploring features which they as individuals are less familiar with. This potentially enables mHealth to be used by physicians in novel ways/for unanticipated emerging patient problems. Therefore, in post-adoptive scenarios physicians may employ different features to cope with changing working requirements.

## Conclusion

This article answers calls for the development of a specific mHealth evaluation framework which is scant in existing literature [8–11]. In meeting this request, we have also addressed the dearth of research examining mHealth in a post-adoptive scenario and its impact upon Perceived Quality of Care Delivery (PQoC).

MHealth presents healthcare organisations with a significant amount of opportunity which benefits healthcare professionals and patients alike. This study informs hospitals and software vendors as to the performance of mHealth by clearly demonstrating that physicians using mHealth at the point-of-care enhances their PQoC a patient receives. As the availability of mHealth continues to increase, we call that all mHealth should be reviewed by clinical experts in order to safeguard the quality of care patients receive.

This study also contributes to the practitioner community by highlighting the importance of adapting mHealth to adhere to users work practices, without unnecessary disruption to the use of the service. Changes in work practices within healthcare environments are often dictated by external forces (e.g. pharmaceutical society introduces new guidelines for dispensing drugs). For PQoC to remain constant, it is imperative that mHealth continuously evolves and adapts to changing work practices and that mHealth be designed with work practices in mind. Indeed, our findings reveal that once technology is embedded, technology characteristics are a secondary consideration for physicians.

Although this research achieved its objective, the results of this study should be interpreted in the context of its limitations. First, this model was examined from a healthcare physician perspective. While a healthcare physician population was appropriate for this study, the conceptual model (see Fig. 1) could be tested across a wide cohort of medical professions (e.g. nurses, physiotherapists, dieticians and, pharmacists). Such context extensions are argued ([88]) p.103) to be “part of on-going efforts to provide generalised measures of TTF constructs”. Additionally, healthcare services are often delivered across different levels (e.g. primary, secondary and territory) and scenarios (e.g. preventive care, urgent care, emergency care, home health, and long-term care) ( [89] p.66). As a result, we further urge future research to also consider these domains. Moreover, individuals use mobile technology, especially smartphones, for both hedonic and utilitarian purposes [90]. Building from this, future research should examine medical professionals who use smartphones which are consumed for both work and personal purposes. Although rich data was obtained from participants in the study to develop and validate the conceptual model, future research could conduct similar empirical work with a larger study population. This will further validate the research model.

## Supplementary information

**Additional file 1.**

**Additional file 2.**

## Data Availability

All data generated or analyzed during this study are included in this published article [and its supplementary information files].

## Abbreviations

H: Hypothesis
IT: Information Technology
MHealth: Mobile health
PLS: Partial Least Squares
PQoC: Perceived quality of care delivery
SEM: Structural Equation Modeling
TTF: Task-technology fit
