# Supplementary material for: MHealth and perceived quality of care delivery: a conceptual model and validation
Source: BMC Med Inform Decis Mak. 2020 Feb 27;20:41. doi: 10.1186/s12911-020-1049-8 (PMC7045642; doi:10.1186/s12911-020-1049-8)
Supplement: Supplementary file 1 — Additional file 1. [file 12911_2020_1049_MOESM1_ESM.docx]

MHealth and Perceived Quality of Care Delivery: A Conceptual Model and Empirical Validation

# Additional Material

| Item description - Measured using 5 point Likert Scale  (Strongly Disagree – Strongly Agree) |
| --- |

| Construct | Description | Item | Item Description | Adapted from: |
| --- | --- | --- | --- | --- |
| Locatability  (Loc) | Availability of mHealth to assist physicans delivery healthcare services. | Loc1 | I have no difficulty findings an mHealth to use when required. | [1] |
|  |  | Loc2 | When providing healthcare services, availability of mHealth is not a problem. |  |
|  |  | Loc3 | There are sufficient amounts of mHealth for me to use in the department in which I am predominantly located. |  |
| Timeliness  (T) | Importance with which a task needs to be performed promptly (urgency). | T1 | In emergency situations, I use mHealth to access patient information. | [1] |
|  |  | T2 | In urgent situations, I use mHealth to help me make clinical decisions. |  |
|  |  | T3 | Timeliness, in terms of accessing relevant patient information through mHealth, is a critical element in urgent situations. |  |
| Reliability  (Rel) | mHealth operates consistently and predictably. | Rel1 | The mHealth is very reliable. | [2] |
|  |  | Rel2 | The mHealth is extremely dependable. |  |
|  |  | Rel3 | The mHealth does not malfunction for me. |  |
| Ease of Use  (EU) | Using mHealth is free of effort. | EU1 | Using the mHealth makes it easier to provide healthcare services. | [3] |
| Learning/  Training  (Learn) |  | Learn1 | In my experience using mHealth encourages me to follow clinical guidelines/protocol. | [3, 4] |
|  |  | Learn2 | Accessing medical reference resources through mHealth help me learn more about delivering healthcare services to patients |  |
|  |  | Learn3 | Intervention alerts (e.g. drug-drug, drug-allergy interactions) when using mHealth help me learn more about delivering healthcare services to patients. |  |
|  |  | Learn4 | mHealth are a convenient source of information or means of communication that assist me with medical learning. |  |
| Relationship with Users/  Functionality  (Fun) | mHealth have the capacity or capability to complete a required task. | Fun1 | The mHealth has the functionality I need. | [5] |
|  |  | Fun2 | The mHealth has the features I require. |  |
|  |  | Fun3 | The mHealth has the ability to do what I want it to do. |  |
| Task Characteristics  (Task) | Defined in the main paper – Hypotheses Development Section. | Task1 | I need to process information from many sources. (non-routineness) | [1, 6] |
|  |  | Task 2 | For me I need to share patient information with other healthcare professionals. (interdependability) |  |
|  |  | Task 3 | For my tasks I require accurate and timely information from other healthcare professionals. (accuracy and time criticality) |  |
| Technology Characteristics  (Tech) | Defined in the main paper – Hypotheses Development Section. | Tech 1 | I use the data accessed through mHealth to support me when delivering healthcare services anytime, anywhere (mobility). | [1, 6] |
|  |  | Tech 2 | I use the data accessed through mHealth to organize which patients I meet first (personalization). |  |
|  |  | Tech 3 | I use the data accessed through mHealth to coordinate the delivery of healthcare services (coordination). |  |
| Self-Efficacy  (SE) | Defined in the main paper – Hypotheses Development Section. | SE1 | I have the necessary skills for using mHealth. | [7] |
|  |  | SE2 | I am self-assured about my capabilities to use the mHealth. |  |
|  |  | SE3 | I am confident in my ability to use the mHealth. |  |
| Routine Use  (RU) | Defined in the main paper – Hypotheses Development Section. | RU1 | The use of mHealth has become a habit for me. | [8] |
|  |  | RU2 | Using the mHealth has become automatic to me. |  |
|  |  | RU3 | The use of the mHealth has become a routine practice when providing healthcare services. |  |
| Feature Use  (FU) | Defined in the main paper – Hypotheses Development Section. | FU1 | I use all of the capabilities offered through mHealth. | [5] |
|  |  | FU2 | I use most of the available features on the mHealth. |  |
|  |  | FU3 | I only use a limited amount of the available features offered through mHealth. |  |
| Value-Adding Use  (VAU) | Defined in the main paper – Hypotheses Development Section. | VAU1 | I explore the features of mHealth (e.g. exploring medical reference resources). | [9] |
|  |  | VAU2 | I often search for new medical/clinical information through mHealth (outside of the primary application). | [3] |
|  |  | VAU3 | I use the mHealth in novel ways. | [10] |
| Performance (PQoC) | Defined in the main paper – MHealth and Perceived Quality of Care Delivery Section. | PQoC1 | In my experience using mHealth increases the quality of patient care. | [3] |
|  |  | PQoC2 | Using the mHealth helps improve the diagnosis of patients. | [11, 12] |
|  |  | PQoC3 | Using the mHealth helps improve the treatment of patients. |  |
|  |  | PQoC4 | Using the mHealth helps improve the monitoring and management of disease within the hospital. |  |
|  |  | PQoC5 | The mHealth supports me in interacting with patients when they request more information. | [3] |

The survey had a total of 40 items which align with the conceptual model presented in the paper. Research [13] argues that one advantage of using these pre-existing questions is that they will have been extensively tested at the time of first use. Using pre-existing items is further argued to be good practice in health, social and behavioural research [14].

**Additional Material – References**

1. Gebauer, J., M.J. Shaw, and M.L. Gribbins, *Task-technology fit for mobile information systems.* Journal of Information Technology, 2010. **25**(3): p. 259-272.

2. McKnight, D.H., *Trust in information technology.* The Blackwell encyclopedia of management, 2005. **7**: p. 329-331.

3. Junglas, I., C. Abraham, and R.T. Watson, *Task-technology fit for mobile locatable information systems.* Decision Support Systems, 2008. **45**(4): p. 1046-1057.

4. DesRoches, C.M., et al., *Electronic health records in ambulatory care—a national survey of physicians.* New England Journal of Medicine, 2008. **359**(1): p. 50-60.

5. Ramamurthy, K., A. Sen, and A.P. Sinha, *Data warehousing infusion and organizational effectiveness.* IEEE Transactions on Systems, Man, and Cybernetics-Part A: Systems and Humans, 2008. **38**(4): p. 976-994.

6. Sun, H., Y. Fang, and H.M. Zou, *Choosing a fit technology: Understanding mindfulness in technology adoption and continuance.* Journal of the Association for Information Systems, 2016. **17**(6): p. 377.

7. Ng, E.H. and H.W. Kim, *Investigating Information systems infusion and the moderating role of habit: A user empowerment perspective.* ICIS 2009 Proceedings, 2009: p. 137.

8. Limayem, M., S.G. Hirt, and C.M. Cheung, *How habit limits the predictive power of intention: The case of information systems continuance.* MIS quarterly, 2007. **31**(4): p. 705-737.

9. Saeed, K.A. and S. Abdinnour-Helm, *Examining the effects of information system characteristics and perceived usefulness on post adoption usage of information systems.* Information & Management, 2008. **45**(6): p. 376-386.

10. Po-An Hsieh, J. and W. Wang, *Explaining employees' extended use of complex information systems.* European Journal of Information Systems, 2007. **16**(3): p. 216 - 227.

11. Pinnock, H., et al., *Professional and patient attitudes to using mobile phone technology to monitor asthma: questionnaire survey.* Primary care respiratory journal, 2006. **15**(4): p. 237.

12. Katz, J.E. and R.E. Rice, *Public views of mobile medical devices and services: A US national survey of consumer sentiments towards RFID healthcare technology.* International journal of medical informatics, 2009. **78**(2): p. 104-114.

13. Hyman, L., J. Lamb, and M. Bulmer. *The use of pre-existing survey questions: Implications for data quality*. in *Proceedings of the European Conference on Quality in Survey Statistics*. 2006.

14. Boateng, G.O., et al., *Best practices for developing and validating scales for health, social, and behavioral research: a primer.* Frontiers in public health, 2018. **6**.
