# Supplementary material for: MHealth and perceived quality of care delivery: a conceptual model and validation
Source: BMC Med Inform Decis Mak. 2020 Feb 27;20:41. doi: 10.1186/s12911-020-1049-8 (PMC7045642; doi:10.1186/s12911-020-1049-8)
Supplement: Supplementary file 2 — Additional file 2. [file 12911_2020_1049_MOESM2_ESM.docx]

MHealth and Perceived Quality of Care Delivery: A Conceptual Model and Empirical Validation

# Additional Material

|  | **EU** | **FU** | **Fun** | **Learn** | **Loc** | **PQoC** | **RU** | **Rel** | **SE** | **T** | **VAU** | **Task** | **Tech** |
| --- | --- | --- | --- | --- | --- | --- | --- | --- | --- | --- | --- | --- | --- |
| **EU1** | 1.000 | 0.410 | 0.508 | 0.447 | 0.440 | 0.618 | 0.575 | 0.443 | 0.378 | 0.282 | 0.328 | 0.433 | 0.578 |
| **FU1** | 0.340 | **0.771** | 0.350 | 0.226 | 0.402 | 0.359 | 0.343 | 0.309 | 0.396 | 0.175 | 0.298 | 0.189 | 0.362 |
| **FU2** | 0.377 | **0.895** | 0.334 | 0.414 | 0.448 | 0.460 | 0.427 | 0.237 | 0.412 | 0.337 | 0.504 | 0.295 | 0.472 |
| **FU3** | 0.281 | **0.782** | 0.232 | 0.197 | 0.269 | 0.274 | 0.367 | 0.117 | 0.359 | 0.283 | 0.298 | 0.081 | 0.289 |
| **Fun1** | 0.403 | 0.314 | **0.869** | 0.310 | 0.493 | 0.450 | 0.553 | 0.643 | 0.432 | 0.237 | 0.353 | 0.241 | 0.518 |
| **Fun2** | 0.439 | 0.382 | **0.907** | 0.350 | 0.409 | 0.458 | 0.516 | 0.554 | 0.395 | 0.234 | 0.279 | 0.222 | 0.515 |
| **Fun3** | 0.490 | 0.288 | **0.843** | 0.298 | 0.425 | 0.420 | 0.472 | 0.487 | 0.323 | 0.286 | 0.311 | 0.162 | 0.421 |
| **Learn1** | 0.298 | 0.311 | 0.295 | **0.862** | 0.268 | 0.640 | 0.433 | 0.211 | 0.348 | 0.323 | 0.682 | 0.383 | 0.476 |
| **Learn2** | 0.239 | 0.094 | 0.177 | **0.748** | 0.012 | 0.414 | 0.150 | 0.120 | -0.020 | 0.186 | 0.250 | 0.259 | 0.308 |
| **Learn3** | 0.432 | 0.334 | 0.291 | **0.802** | 0.425 | 0.568 | 0.410 | 0.324 | 0.309 | 0.376 | 0.583 | 0.412 | 0.435 |
| **Learn4** | 0.421 | 0.331 | 0.369 | **0.741** | 0.172 | 0.574 | 0.279 | 0.379 | 0.194 | 0.184 | 0.374 | 0.258 | 0.358 |
| **Loc1** | 0.408 | 0.355 | 0.438 | 0.271 | **0.877** | 0.397 | 0.502 | 0.331 | 0.452 | 0.219 | 0.428 | 0.297 | 0.395 |
| **Loc2** | 0.393 | 0.392 | 0.462 | 0.337 | **0.843** | 0.289 | 0.333 | 0.510 | 0.503 | 0.080 | 0.315 | 0.196 | 0.464 |
| **Loc3** | 0.258 | 0.437 | 0.340 | 0.114 | **0.746** | 0.160 | 0.353 | 0.355 | 0.359 | 0.011 | 0.229 | 0.171 | 0.357 |
| **PQoC1** | 0.532 | 0.373 | 0.428 | 0.683 | 0.294 | **0.815** | 0.626 | 0.362 | 0.314 | 0.373 | 0.578 | 0.443 | 0.485 |
| **PQoC2** | 0.422 | 0.360 | 0.388 | 0.619 | 0.338 | **0.814** | 0.492 | 0.330 | 0.230 | 0.327 | 0.515 | 0.380 | 0.529 |
| **PQoC3** | 0.505 | 0.406 | 0.398 | 0.550 | 0.244 | **0.861** | 0.488 | 0.327 | 0.367 | 0.400 | 0.428 | 0.486 | 0.473 |
| **PQoC4** | 0.465 | 0.313 | 0.450 | 0.485 | 0.276 | **0.731** | 0.393 | 0.281 | 0.237 | 0.331 | 0.323 | 0.362 | 0.552 |
| **PQoC5** | 0.534 | 0.359 | 0.359 | 0.460 | 0.292 | **0.761** | 0.548 | 0.198 | 0.316 | 0.335 | 0.572 | 0.411 | 0.405 |
| **RU1** | 0.534 | 0.417 | 0.528 | 0.368 | 0.465 | 0.577 | **0.915** | 0.489 | 0.410 | 0.453 | 0.479 | 0.442 | 0.425 |
| **RU2** | 0.518 | 0.455 | 0.576 | 0.408 | 0.416 | 0.567 | **0.914** | 0.551 | 0.566 | 0.363 | 0.530 | 0.437 | 0.472 |
| **RU3** | 0.522 | 0.403 | 0.508 | 0.390 | 0.459 | 0.621 | **0.906** | 0.410 | 0.487 | 0.388 | 0.552 | 0.442 | 0.482 |
| **Rel1** | 0.382 | 0.241 | 0.551 | 0.284 | 0.406 | 0.288 | 0.494 | **0.897** | 0.463 | 0.179 | 0.213 | 0.235 | 0.347 |
| **Rel2** | 0.424 | 0.285 | 0.605 | 0.300 | 0.443 | 0.370 | 0.553 | **0.927** | 0.543 | 0.316 | 0.328 | 0.389 | 0.451 |
| **Rel3** | 0.380 | 0.197 | 0.570 | 0.322 | 0.432 | 0.353 | 0.357 | **0.857** | 0.301 | 0.152 | 0.180 | 0.211 | 0.347 |
| **SE1** | 0.323 | 0.409 | 0.357 | 0.287 | 0.532 | 0.326 | 0.512 | 0.410 | **0.900** | 0.159 | 0.458 | 0.346 | 0.287 |
| **SE2** | 0.377 | 0.497 | 0.460 | 0.261 | 0.503 | 0.372 | 0.505 | 0.521 | **0.934** | 0.134 | 0.462 | 0.237 | 0.446 |
| **SE3** | 0.336 | 0.393 | 0.388 | 0.260 | 0.432 | 0.311 | 0.447 | 0.426 | **0.908** | 0.170 | 0.408 | 0.265 | 0.394 |
| **T1** | 0.141 | 0.276 | 0.253 | 0.273 | 0.170 | 0.346 | 0.349 | 0.260 | 0.133 | **0.865** | 0.338 | 0.486 | 0.268 |
| **T2** | 0.273 | 0.321 | 0.222 | 0.345 | 0.041 | 0.424 | 0.353 | 0.156 | 0.119 | **0.853** | 0.366 | 0.449 | 0.366 |
| **T3** | 0.276 | 0.214 | 0.239 | 0.246 | 0.158 | 0.321 | 0.385 | 0.206 | 0.166 | **0.754** | 0.423 | 0.580 | 0.232 |
| **VAU1** | 0.336 | 0.422 | 0.395 | 0.548 | 0.460 | 0.451 | 0.527 | 0.350 | 0.445 | 0.358 | **0.802** | 0.278 | 0.550 |
| **VAU2** | 0.205 | 0.396 | 0.218 | 0.518 | 0.258 | 0.557 | 0.477 | 0.114 | 0.295 | 0.415 | **0.841** | 0.342 | 0.417 |
| **VAU3** | 0.279 | 0.333 | 0.286 | 0.505 | 0.304 | 0.514 | 0.415 | 0.229 | 0.473 | 0.357 | **0.843** | 0.346 | 0.441 |

Discriminant Validity – Cross Loading
